# Developing and validating a digital literacy assessment framework for pre-service physical education teachers: a Delphi-AHP study

**DOI:** 10.3389/fpsyg.2026.1852701

**Published:** 2026-08-20

**Authors:** Shuang Zhao, Guoqiang Song, Jiao Wang, Guoyou Qin, Xitang Zhao

**Affiliations:** 1 School of Physical Education, Hanjiang Normal University, Shiyan, Hubei, China; 2 Rattana Bundit University, Bangkok, Thailand; 3 Central China Normal University, Wuhan, Hubei, China; 4 Nanjing Sport Institute, Nanjing, Jiangsu, China

**Keywords:** analytic hierarchy process, assessment framework, Delphi method, digital literacy, physical education teacher education, pre-service physical education teacher

## Abstract

**Background:**

The digital transformation of education has introduced new demands on the professional competencies of physical education (PE) teachers. However, there is a lack of a comprehensive assessment framework specifically designed for pre-service PE teachers. This study aims to develop a scientifically sound digital literacy assessment framework for pre-service PE teachers, clarify the core components of their digital literacy, and promote the sustainable development of PE teacher education.

**Methods:**

The preliminary indicator system was derived from a systematic literature review spanning 2015 to 2026 and policy analysis of DigCompEdu and UNESCO ICT-CFT frameworks. Guided by the TPACK framework and integrating educational psychology theories including Self-Determination Theory, the Technology Acceptance Model, and Teacher Self-Efficacy Theory, the Delphi method was employed with a multidisciplinary expert panel of 20 specialists from physical education pedagogy, educational technology, and educational psychology to systematically assess the indicator system for pre-service PE teachers’ digital literacy. A 5-point Likert scale examined consensus and stability. After two Delphi rounds, the Analytic Hierarchy Process determined indicator weights. Future empirical validation with pre-service PE teachers is recommended to confirm the framework’s applicability and predictive validity.

**Results:**

After two rounds of Delphi surveys, the final framework comprised 4 primary indicators and 12 secondary indicators. The expert authority coefficient was 0.825. Kendall’s coefficient of concordance increased from 0.414 in the first round to 0.568 in the second round (*p* < 0.001). Weight analysis showed that Digitalized Physical Education Teaching Integration Competence had the highest weight (0.3474), followed by Physical Education Digital Tools and Data Literacy (0.3032), Intelligent Technology Cognition and Foundational Literacy (0.1981), and Professional Development and Social Impact (0.1513). All consistency ratios for the judgment matrices were below 0.1, meeting the consistency requirements.

**Conclusion:**

Weight analysis showed that Digitalized Physical Education Teaching Integration Competence had the highest weight (0.3474), followed by Physical Education Digital Tools and Data Literacy (0.3032), Intelligent Technology Cognition and Foundational Literacy (0.1981), and Professional Development and Social Impact (0.1513). The prioritization of integration competence over foundational knowledge highlights the importance of practical application in PE teachers’ digital literacy, confirming that the ability to operationalize technology in teaching is valued above declarative knowledge alone. The framework is scientifically sound and reliable. It provides target guidance for pre-service PE teacher cultivation and a theoretical basis for curriculum reform. Its underlying psychological logic also offers a new perspective for understanding teacher digital literacy development from a motivational and cognitive standpoint. Future empirical validation with pre-service PE teachers is recommended to confirm the framework’s applicability and predictive validity.

## 1 Introduction

In recent years, UNESCO has placed “Quality Physical Education” at the core of school physical education reform, emphasizing its irreplaceable role in fostering the holistic development of individuals (Uhlenbrock and Meier, 2021). In China, policy documents such as the Healthy China 2030 Plan and China’s Education Modernization 2035 have likewise called for deeper integration of information technology with education and for the enhancement of teachers’ digital literacy (Feng and Sumettikoon, 2024; Yan and Yang, 2021). These policy orientations set higher expectations for the professional competence of physical education (PE) teachers and provide the practical background for this study (Royet et al., 2025). Previous studies have identified teachers’ digital literacy as a core competency for professional development in the digital era (Scherer et al., 2019; Wohlfart et al., 2024).

Pre-service PE teachers are those who have received teacher education but have not yet formally entered the profession; they represent the future backbone of school physical education (Mayo-Rota et al., 2025). Digital literacy refers to teachers’ ability to access, understand, integrate, create, and disseminate information in digital environments, encompassing intelligent technology awareness, digital tool operation, data-driven instructional decision-making, and digital professional development (Hammoda and Foli, 2024; Zhao et al., 2025), profoundly transforming instructional models and enabling personalized learning, real-time feedback, and motor skill assessment (Gustian et al., 2026). Against this backdrop, systematically constructing a digital literacy assessment framework for pre-service PE teachers is both a practical necessity to address current challenges and a crucial step to enhance the quality of PE teacher education.

The development of students’ motor skills depends heavily on the professional guidance and pedagogical organization provided by PE teachers (Logan et al., 2012). Research shows that structured PE curricula are significantly more effective than unstructured activities in promoting fundamental motor skill development (Abusleme-Allimant et al., 2023), and the professional competence of PE teachers is one of the most critical factors influencing instructional effectiveness (Marinsek and Kovac, 2018). In the digital era, the design, implementation, and evaluation of structured curricula increasingly rely on digital tools such as motion data analytics and smart devices, which demand a new set of competencies from teachers (Gao et al., 2025), Namely, the ability to integrate digital technology into PE instruction (Caneva et al., 2023). The pre-service stage is a critical period for teachers’ professional development, serving as an important window for the formation of educational beliefs (Heine and König, 2025), teaching efficacy, and professional identity (González-Calvo and Fernández-Balboa, 2018). Cultivating and assessing digital literacy at this stage will profoundly shape their technology integration behaviors after entering the workforce (MacPhail et al., 2025; Zhang et al., 2026).

However, current efforts to cultivate digital literacy among pre-service PE teachers suffer from systemic deficiencies. In terms of cognition and awareness, they generally lack an understanding of the educational value of digital technology and fail to deeply integrate it with PE instruction (Zhong et al., 2025). In skills and application, they exhibit significant shortcomings in using smart devices to collect athletic data, analyzing and interpreting data, and developing digital teaching resources (Attila and László, 2023). In curriculum and cultivation, pre-service PE teacher training programs lack systematic digital literacy education, and curriculum design does not adequately reflect the integration of technology and PE (Saiz-González et al., 2025). In attitudes and beliefs, some pre-service PE teachers hold negative attitudes toward digital technology, exhibit high levels of techno-anxiety, and show low technology acceptance, which directly affects their adaptability and teaching effectiveness in digital learning environments (Dong and Sheng, 2026). These practical issues indicate that cultivating digital literacy involves far more than technical operation; it is closely tied to the cognitive and motivational mechanisms that underpin effective technology integration.

A substantial body of research confirms that teachers’ digital literacy is a key factor influencing the effectiveness of digital teaching (Zhang and Wu, 2025). Teacher teaching efficacy is a critical variable influencing teaching behavior and learning outcomes (Huang et al., 2025), and in the digital context, teachers’ technology self-efficacy directly affects their technology integration (Zeng et al., 2022). From a cognitive psychology perspective, effective digital teaching requires data-driven decision-making—involving information perception, encoding, comparison, and reasoning—which is closely linked to teachers’ metacognitive skills (Alhadad, 2018; Şen, 2023), Yet cognitive capacity alone is insufficient; motivation matters equally. The Technology Acceptance Model (TAM) highlights perceived usefulness and ease of use as the core cognitive predictors of technology adoption (Phelps et al., 2021). Meanwhile, Self-Determination Theory (SDT) emphasizes that satisfying the basic psychological needs for autonomy, competence, and relatedness fosters the internalization of external norms and active engagement in digital learning (Sun et al., 2017). When pre-service teachers experience autonomy, competence, and belonging in digital learning contexts, they are more likely to internalize technology as part of their teaching practice (Şahin and Şahin, 2022). This motivational-cognitive mechanism forms the psychological foundation for effectively integrating digital technology into PE teaching.

**FIGURE 1:**
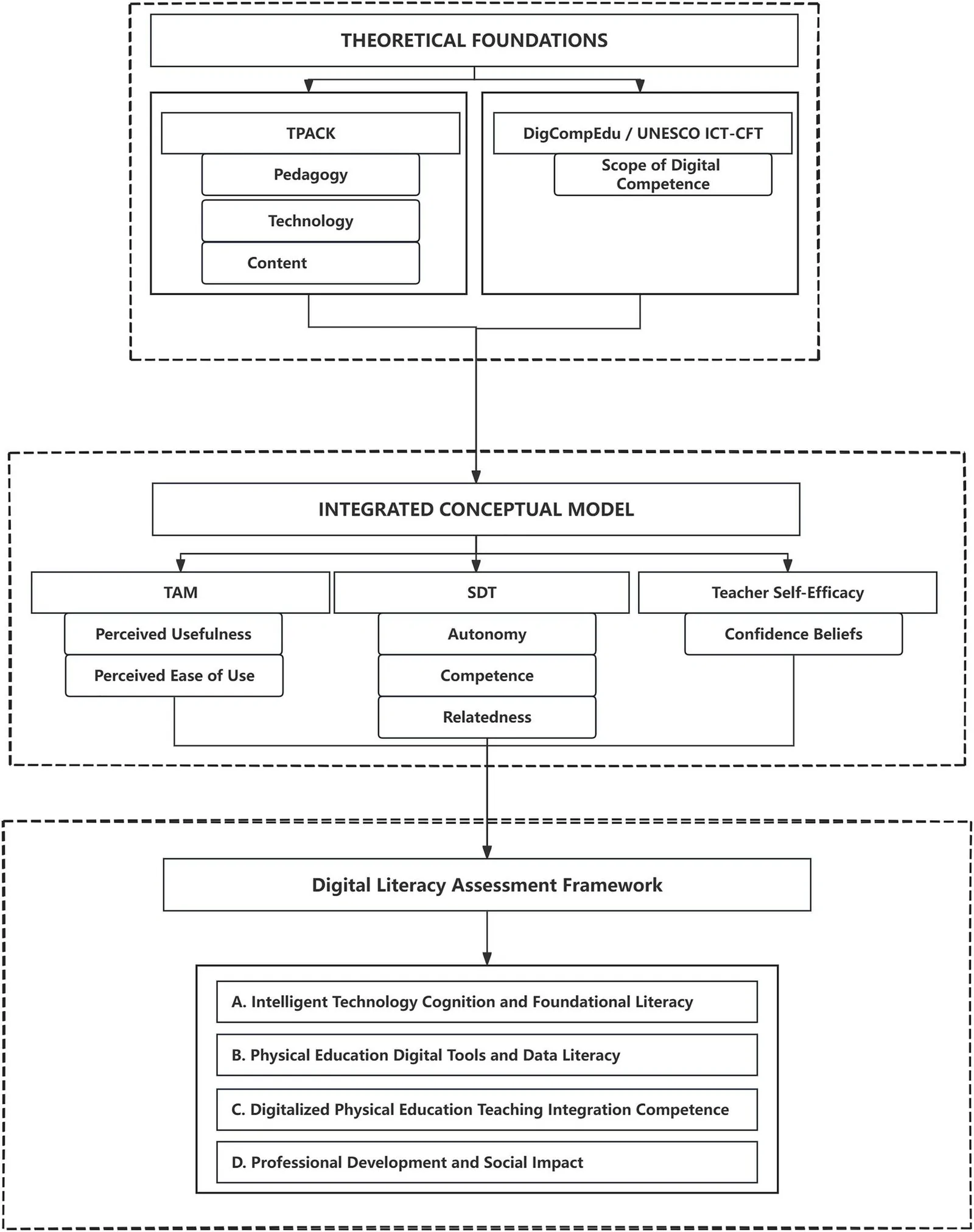
Conceptual model illustrating the integration of TPACK, DigCompEdu, UNESCO ICT-CFT, SDT, TAM, and teacher self-efficacy theories and their mapping to the four primary dimensions of the digital literacy assessment framework. The upper section depicts the theoretical foundations, including TPACK and DigCompEdu/UNESCO ICT-CFT; the middle section presents the motivational-cognitive mechanisms from TAM, SDT, and self-efficacy that drive competence enactment; and the lower section shows the final output—the four-dimensional framework.

To systematically operationalize these theoretical underpinnings, we propose an integrated conceptual model (Figure 1). In this model, the TPACK framework provides the pedagogical-technological-content knowledge structure that specifies what competencies are needed. While the DigCompEdu and UNESCO ICT-CFT frameworks (Redecker, 2017; UNESCO, 2018) offer complementary guidance on the scope of digital literacy for educators. The motivational-cognitive mechanisms derived from the Technology Acceptance Model, Self-Determination Theory, and teacher self-efficacy research—specifically perceived usefulness and ease of use, the needs for autonomy, competence, and relatedness, and confidence beliefs—serve as the driving forces that determine how these competencies are enacted. Digital literacy, therefore, is not merely a set of technical skills; it is activated when teachers perceive digital tools as useful, feel psychologically capable of using them, and have confidence in their own teaching effectiveness. This integrated framework guided our indicator development and weight interpretation.

The practical problems described above highlight the urgent need for a systematic assessment tool for pre-service PE teachers’ digital literacy. However, a review of the existing literature reveals that although numerous studies have explored the components and assessment methods of teacher digital literacy, such as the DigCompEdu framework (Momdjian et al., 2025; Palacios-Rodríguez et al., 2025; Rutten and Brouwer-Truijen, 2025), research in physical education has largely focused on traditional teaching competence or broader professional literacy (Baumgartner, 2022), with limited attention to digital literacy. More specifically, three gaps can be identified: first, a lack of systematic studies specifically targeting the digital literacy of pre-service PE teachers (Østerlie et al., 2025); second, insufficient in-depth exploration of the internal structure of digital literacy from a psychological mechanism perspective (Reichert et al., 2022);and third, a lack of a comprehensive assessment framework specifically designed for pre-service PE teachers and validated by experts with assigned weights. Although a few studies have begun to explore assessment frameworks for PE teachers’ digital or online learning literacy (Tian et al., 2024), these frameworks predominantly target in-service teachers, leaving a systematic tool for pre-service PE teachers notably absent.

Accordingly, this study aimed to: (1) develop a scientific, systematic, and operational digital literacy assessment framework for pre-service PE teachers from a psychological mechanism perspective; (2) refine the indicator structure through two rounds of Delphi expert consultation (Dalkey et al., 1969), drawing on systematic literature review and policy analysis; (3) determine indicator weights using the Analytic Hierarchy Process (AHP) (Saaty and Vargas, 1987), and thereby ensure the framework’s scientific rigor and practical applicability. The resulting framework is intended to provide clear target guidance for pre-service PE teacher cultivation, a theoretical basis for curriculum reform, and a practical reference for teacher training and teaching evaluation in PE teacher education programs, ultimately promoting the professional development of physical education in the digital age.

## 2 Materials and methods

### 2.1 Research design

**FIGURE 2:**
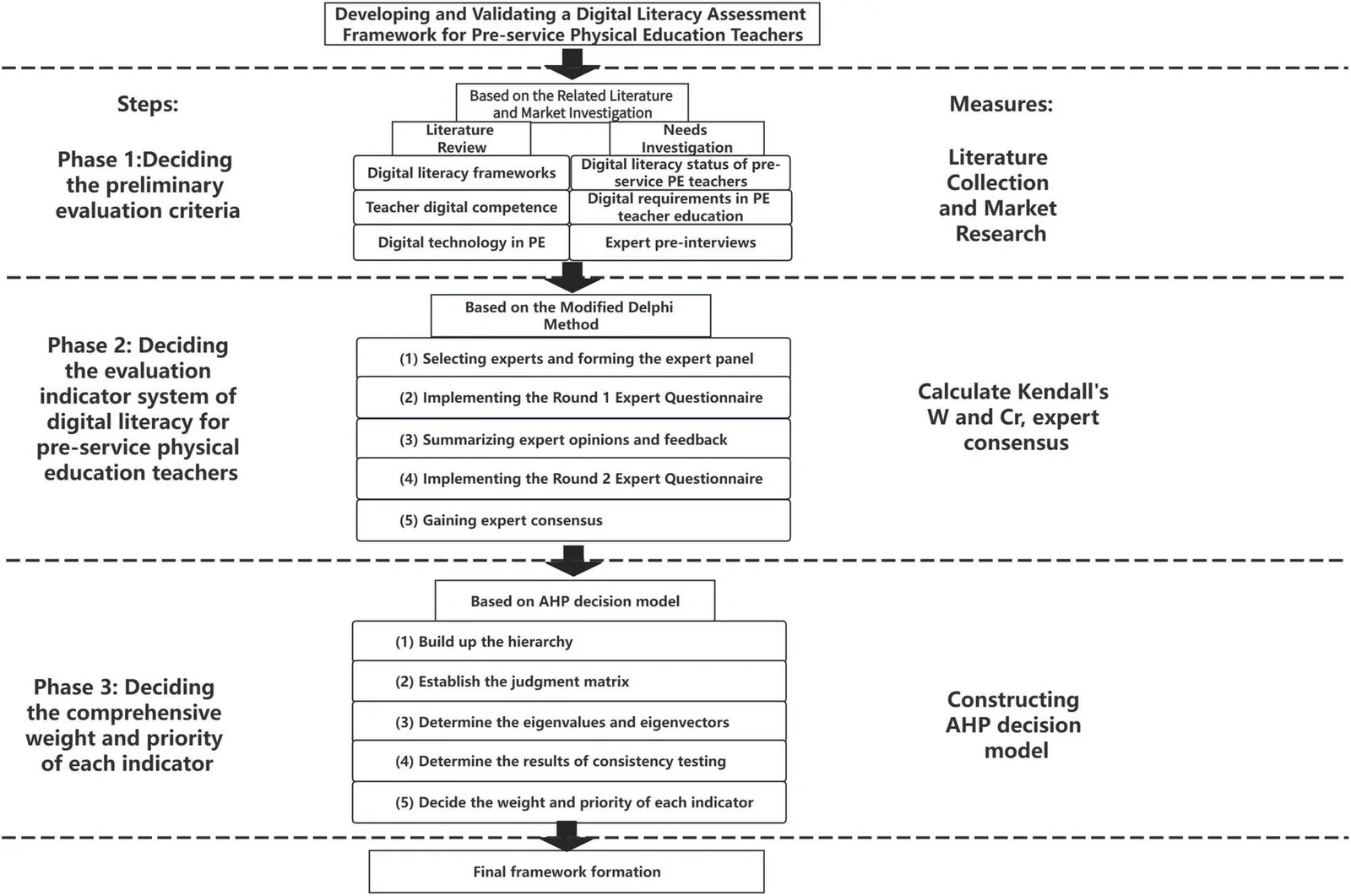
Research process flowchart of this study. The process comprises four stages: systematic literature review (PRISMA guidelines) for preliminary indicator generation; two rounds of Delphi consultation (20 experts, Round 1: ≥ 80% agreement, Round 2: mean > 3.5, CV < 0.25, full-score > 0.2) for indicator refinement; AHP weight determination using the geometric mean method; and final framework formation. All AHP consistency ratios were below 0.1.

This study employed a combination of the Delphi method (Dalkey et al., 1969) and the Analytic Hierarchy Process (AHP) (Saaty and Vargas, 1987) to construct a digital literacy assessment framework for pre-service physical education teachers. The research process comprised four stages: (1) constructing a preliminary indicator system through literature review; (2) conducting two rounds of Delphi expert consultation to screen and optimize indicators; (3) applying AHP to determine indicator weights; and (4) aggregating the results to form the final framework. The overall research process is shown in Figure 2.

### 2.2 Construction of the preliminary indicator system

We conducted a systematic literature search following PRISMA guidelines. Databases: Web of Science, Scopus, and CNKI (Chinese National Knowledge Infrastructure). Time span: January 2015 to March 2026. Search terms were: (“digital literacy” OR “digital literacy” OR “digital literacy” OR “ICT literacy”) AND (“physical education” OR “PE teacher” OR “pre-service teacher” OR “teacher education”) AND (“assessment” OR “framework” OR “indicator” OR “evaluation”). Inclusion criteria: (1) peer-reviewed empirical or theoretical studies; (2) studies addressing teacher digital literacy, with or without a PE focus; (3) official policy frameworks (DigCompEdu, UNESCO ICT-CFT). Exclusion criteria: (1) non-English or non-Chinese publications; (2) studies focusing solely on student digital literacy without teacher implications; (3) conference abstracts or book reviews. The initial search yielded 347 records. After removing duplicates (*n* = 89) and screening titles/abstracts (*n* = 258), we retained 127 full-text articles and 5 policy documents for in-depth analysis. Two researchers independently extracted candidate indicators using thematic analysis, with disagreements resolved through discussion. We initially identified 56 candidate items, which were then grouped into four broad themes based on the TPACK framework (Technology, Pedagogy, Content, and their intersections) and the DigCompEdu areas. An advisory panel of three senior PE educators reviewed the grouped items for clarity and relevance, merging overlapping items and removing those irrelevant to pre-service PE contexts. This process resulted in the preliminary framework of 4 primary and 12 secondary indicators (see Supplementary Table 1 for the full initial list and sources).

The preliminary indicator structure was directly derived from the above conceptual model: each primary dimension corresponds to a core theoretical component. For example, Dimension A (Intelligent Technology Cognition and Foundational Literacy) directly reflects TAM’s cognitive beliefs, Dimension C (Digitalized Physical Education Teaching Integration Competence) embodies the TPACK core, and Dimension D (Professional Development and Social Impact) addresses SDT’s relatedness need.

### 2.3 Expert selection

Expert selection is a critical criterion for ensuring the validity of Delphi studies The sample size of 20 was determined based on Delphi methodological guidelines, which recommend 15-25 experts to achieve reliable consensus while maintaining manageability (Grant and Khodyakov, 2025). To ensure multi-disciplinary representation, we purposively recruited experts from three relevant fields: 10 from physical education pedagogy (to cover subject-specific teaching knowledge), 6 from educational technology (to provide technical and digital literacy expertise), and 4 from educational psychology (to address motivational and cognitive mechanisms). This stratification was deliberately designed to balance the pedagogical, technical, and psychological perspectives that underpin our theoretical framework. The basic characteristics of the experts are shown in Table 1. Prior to the formal Delphi rounds, the same panel participated in semi-structured interviews to provide preliminary feedback on the indicator structure, which informed the development of the Round 1 questionnaire.

**TABLE 1:** Basic characteristics of experts.

Item	Category	Count	Percentage (%)
Gender	Male	12	60
	Female	8	40
Age	30–39 years	4	20
	40–49 years	9	45
	50 years and above	7	35
Education	Master’s	12	60
	Ph.D.	8	40
Professional Title	Associate senior	11	55
	Senior	9	45
Field of expertise	Physical education	10	50
	Educational technology	6	30
	Educational psychology	4	20
Years of experience	10–19 years	6	30
	20–29 years	9	45
	30 years and above	5	25

### 2.4 Delphi method implementation

#### 2.4.1 Questionnaire design and scoring method

The questionnaire comprised four parts: (1) research background, purpose, and instructions; (2) an evaluation table for pre-service PE teacher digital literacy indicators, using a 5-point Likert scale (Czekanski et al., 2025) requiring experts to rate the importance of each indicator: 5 = very important, 4 = important, 3 = moderate, 2 = unimportant, 1 = very unimportant; (3) a basic information survey; (4) a survey on expert familiarity and judgment basis. Data analysis was performed using Excel and SPSS software. The indicator screening criteria were: mean > 3.5, coefficient of variation < 0.25, full-score frequency > 0.2 (Hohmann et al., 2025; Zartha Sossa et al., 2019). Indicators meeting all three criteria were retained; otherwise, they were deleted. Open-ended questions were included to collect suggestions for modification, addition, or deletion of indicators.

#### 2.4.2 Expert positive coefficient

The expert positive coefficient was reflected by the questionnaire response rate. A higher response rate indicates greater expert interest and engagement in the study (Chen et al., 2022).

#### 2.4.3 Expert authority

The expert authority coefficient (Cr) was calculated as the arithmetic mean of the expert judgment basis (Ca) and the expert familiarity coefficient (Cs), i.e., Cr equals the sum of Cs and Ca divided by two (Hasson et al., 2000). The judgment basis included four dimensions: theoretical analysis, practical experience, knowledge from domestic and international peers, and intuition. Familiarity was rated on a 5-point scale (very familiar = 1.0, familiar = 0.8, moderate = 0.6, not very familiar = 0.4, unfamiliar = 0.2). When Cr was greater than or equal to 0.7, the expert consultation results were considered to have high credibility (Min et al., 2024).

#### 2.4.4 Expert coordination

The coefficient of variation (CV) and Kendall’s coefficient of concordance (W) were used to assess the coordination of expert opinions. A smaller CV indicates less divergence of opinion among experts on each indicator. The W value ranges from 0 to 1, with larger values indicating higher coordination. A *p* < 0.05 for the W statistic indicates a statistically significant degree of consensus among expert ratings (Zhang et al., 2025).

#### 2.4.5 Consensus criteria

This study employed two rounds of Delphi consultation to achieve expert consensus. In Round 1, we used a relatively liberal retention criterion to avoid prematurely discarding potentially relevant indicators: at least 80% of experts rated the indicator as ≥ 4 (agree or strongly agree). In Round 2, after receiving the first-round feedback, we applied a stricter multi-criterion standard to confirm high-level consensus: (1) mean > 3.5; (2) CV < 0.25; (3) full-score frequency > 0.2. The rationale for tightening the criteria was to ensure that only indicators with strong and consistent expert support were included in the final framework, as recommended by Zartha Sossa et al. (2019). After Round 1, the following changes were made (details in Supplementary Table 2): – Deleted: B1 “Smart Sports Equipment Operation” (failed all three retention criteria: mean = 3.35, CV = 0.261, full-score frequency = 0.10). – Added: B11 “Sports Data Collection and Processing” (suggested by multiple experts who noted the growing use of wearable devices and motion sensors in PE, requiring data collection skills). – Refined wording: C2 was clarified to explicitly mention “simulated or real practicum settings” based on expert feedback. All other 11 secondary indicators met the retention criteria and were retained. No additional modifications were suggested in Round 2.

### 2.5 Analytic hierarchy process

#### 2.5.1 Construction of the hierarchical structure model

Based on the indicator system established through the Delphi method, this study transformed it into a hierarchical evaluation system for AHP. The division of primary indicators was primarily based on the following theoretical frameworks: (1) International Teacher Digital Literacy Frameworks: the DigCompEdu framework (García-Vandewalle García et al., 2023; Santos et al., 2025) and the TPACK framework (Park et al., 2025; Pérez-Fernández and Ladrón-de-Guevara, 2024); (2) Domestic Teacher Digital Literacy Standards: the “Teacher Digital Literacy” standard (JY/T 0646-2022) released by the Ministry of Education of China in 2022 (Liu et al., 2025); (3) Characteristics of the Physical Education Discipline (Potop et al., 2023; Wang and Yang, 2025).

#### 2.5.2 Construction of judgment matrices

Using the 1–9 scaling method (Saaty and Vargas, 1987), experts were asked to perform pairwise comparisons of indicators within the same level to construct judgment matrices. In this study, 20 experts were invited to complete judgment matrices. For the pairwise comparisons, we provided each expert with a structured questionnaire (see Supplementary Questionnaire 3). Experts were asked to compare indicators within each group (e.g., the four primary indicators) using the 1-9 Saaty scale. To aggregate the 20 individual judgment matrices, we employed the geometric mean method (Pant et al., 2022), which is recommended for synthesizing group decisions in AHP because it preserves the reciprocal property of the matrices. All calculations were performed using Yaahp 12.9 software. The aggregated judgment matrix for the primary indicators is provided in Supplementary Table 5, and individual expert consistency ratio (CR) values are reported in Supplementary Table 3. To assess the robustness of the AHP-derived weights, we compared the weights derived from the geometric mean method with those from the arithmetic mean. The rank order of the top two primary indicators remained identical (C > B), and the overall weight differences were within acceptable ranges. All 20 individual consistency ratios (CR) across all groups were below 0.1 (see Supplementary Table 3 for a complete list), confirming the consistency of the expert judgments. These results suggest that the AHP weight estimates are robust to the method of aggregation.

#### 2.5.3 Consistency check

The consistency ratio (CR) was calculated to verify the consistency of the judgment matrices. When CR < 0.1, the judgment matrix was considered to have satisfactory consistency (Saaty and Vargas, 1987). For individual expert consistency, we computed the CR for each matrix. The CR values for all 20 experts across all groups were below 0.1 (see Supplementary Table 3 for a complete list). No individual matrix exceeded the 0.1 threshold; therefore, no matrices were excluded or required re-evaluation. The average random consistency index RI values are shown in Table 2.

**TABLE 2:** Average random consistency index RI values for judgment matrices.

Matrix order	1	2	3	4	5	6	7	8	9	10	11	12
RI	0	0	0.52	0.89	1.12	1.26	1.36	1.41	1.46	1.49	1.52	1.54

### 2.6 Statistical analysis

Data entry and organization were performed using Excel software. Statistical indicators such as mean, standard deviation, coefficient of variation, full-score frequency, and Kendall’s coefficient of concordance were calculated using SPSS 28.0 software. The AHP calculations were performed with the assistance of Yaahp 12.9 software.

## 3 Results

### 3.1 Expert positive coefficient and authority

Both Delphi rounds achieved a 100% response rate, with all 20 questionnaires returned (Table 3). The expert authority coefficient (Cr) was calculated as the arithmetic mean of the expert judgment basis (Ca) and the familiarity coefficient (Cs), i.e., Cr equals the sum of Cs and Ca divided by two (Jiang et al., 2020). The quantitative calculations for expert familiarity and judgment basis are shown in Tables 4, 5.

**TABLE 3:** Questionnaire response rates.

Round	Number sent	Number returned	Expert positive coefficient
First round	20	20	100%
Second round	20	20	100%

**TABLE 4:** Calculation of expert familiarity coefficient.

Familiarity level	Unfamiliar	Not very familiar	Moderately familiar	Familiar	Very familiar
Number of experts	0	2	4	8	6
Weight	0.2	0.4	0.6	0.8	1
Familiarity coefficient (Cs)	Cs = 0.78				

**TABLE 5:** Calculation of expert judgment basis coefficient.

Judgment basis	High		Medium		Low		Score
	n	Weight	n	Weight	n	Weight	
Practical experience	12	0.5	6	0.4	2	0.3	0.45
Theoretical analysis	8	0.3	8	0.2	4	0.1	0.22
Knowledge from peers	4	0.1	10	0.1	6	0.1	0.10
Intuition	2	0.1	6	0.1	12	0.1	0.10
Judgment basis coefficient (Ca)							0.87

Based on the data from Table 4 and in this table, the expert familiarity coefficient (Cs) was 0.78, and the judgment basis coefficient (Ca) was 0.87. Therefore, the expert authority coefficient Cr = (0.78+0.87)/2 = 0.825, which is greater than 0.7.

### 3.2 Delphi consultation results

The results of the first round of consultation showed that all 4 primary indicators met the retention criteria. Among the 12 secondary indicators, B1 (Smart Sports Equipment Operation) did not meet the screening criteria and was therefore deleted. Based on expert suggestions, B11 (Sports Data Collection and Processing) was added. Experts noted that with the proliferation of wearable devices and motion sensors in physical education, teachers need the ability to collect and process student motor data, which is the foundation for data-driven teaching (Zhao and Su, 2026). This indicator, together with the original B2 and B3, constituted the B category for the second round of consultation. Except for B1, the remaining 11 secondary indicators all met the retention criteria.

The results of the second round of consultation showed that all 12 secondary indicators (including the newly added B11) met the screening criteria. No other modifications were suggested by the experts. The detailed statistical results from the two rounds of Delphi consultation are summarized in Table 6. The detailed descriptive statistics for all indicators across both rounds, including mean, standard deviation, coefficient of variation, and full-score frequency, are provided in Supplementary Table 4.

**TABLE 6:** Summary of Delphi consultation results.

Indicator	Round 1			Round 2		
	Mean	CV	Full-score freq (%)	Mean	CV	Full-score freq (%)
A. Intelligent technology cognition and foundational literacy	4.7	0.1215	0.75	–	–	–
A1. Intelligent technology cognition	3.9	0.2185	0.30	4.1	0.2224	0.45
A2. Digital mindset and awareness	4.7	0.1398	0.80	4.85	0.0755	0.85
A3. Digital ethics and safety preparedness	4.6	0.1639	0.75	4.7	0.1215	0.75
B. Physical education digital tools and data literacy	4.25	0.1850	0.45	–	–	–
B1. Smart sports equipment operation	3.35	0.2612	0.10	–	–	–
B11. Sports data collection and processing	–	–	–	4.55	0.1122	0.55
B2. Sports data analysis capability	4.65	0.1443	0.75	4.1	0.1922	0.35
B3. Sports Digital Resource Development	4.05	0.2190	0.40	4.95	0.0452	0.95
C. Digitalized physical education teaching integration competence	4.15	0.2109	0.45	–	–	–
C1. Smart physical education lesson design	4.0	0.1814	0.25	4.9	0.0628	0.90
C2. Smart physical education classroom simulation implementation	4.65	0.1443	0.75	3.95	0.1922	0.25
C3. Data-driven teaching evaluation	4.8	0.0855	0.80	3.8	0.2193	0.25
D. Professional development and social impact	4.60	0.1479	0.70	—	—	—
D1. Digitalized professional learning	3.85	0.2273	0.30	4.75	0.1158	0.80
D2. Digital teaching research collaboration participation	3.85	0.2273	0.30	4.9	0.0628	0.90
D3. Digital social responsibility fulfillment	4.65	0.1443	0.75	4.1	0.2079	0.40

After two rounds of Delphi expert consultation, the final constructed digital literacy assessment framework for pre-service physical education teachers consists of 4 primary indicators and 12 secondary indicators. Its hierarchical structure model is shown in Table 7.

**TABLE 7:** Hierarchical structure model.

Goal level	Primary indicator	Secondary indicator
G. Digital literacy of pre-service physical education teachers	A. Intelligent technology cognition and foundational literacy	A1. Intelligent technology cognition
		A2. Digital mindset and awareness
		A3. Digital ethics and safety preparedness
	B. Physical education digital tools and data literacy	B1. Sports data collection and processing
		B2. Sports data analysis capability
		B3. Sports digital resource development
	C. Digitalized physical education teaching integration competence	C1. Smart physical education lesson design
		C2. Smart physical education classroom simulation implementation
		C3. Data-driven teaching evaluation
	D. Professional development and social impact	D1. Digitalized professional learning
		D2. Digital teaching research collaboration participation
		D3. Digital social responsibility fulfillment

The division of primary indicators was based on the DigCompEdu framework, the TPACK framework, China’s Ministry of Education “Teacher Digital Literacy” industry standard (JY/T 0646-2022), and the characteristics of the physical education discipline.

### 3.3 Expert coordination

In the first round of expert consultation, the coefficient of variation for primary indicators ranged from 0.1215 to 0.2109, with a Kendall’s W of 0.414 (*p* < 0.001); for secondary indicators, the CV ranged from 0.0855 to 0.2612, with a Kendall’s W of 0.451 (*p* < 0.001). In the second round, the CV range for secondary indicators narrowed to 0.0452–0.2224, and the Kendall’s W increased to 0.568 (*p* < 0.001), as detailed in Table 8.

**TABLE 8:** Coordination of expert opinions.

Evaluation content	CV Range	Kendall’s W	Chi-square	df	*p*-value
Round 1					
Primary indicators	0.1215–0.2109	0.414	24.818	3	< 0.001
Secondary indicators	0.0855–0.2612	0.451	99.113	11	< 0.001
Round 2					
Secondary indicators	0.0452–0.2224	0.568	125.03	11	< 0.001

### 3.4 Analytic hierarchy process results

#### 3.4.1 Primary indicator weights

The geometric mean method was used to aggregate the judgment matrices from the 20 experts, resulting in the integrated matrix and weights for the primary indicators. Among the primary indicators, the weight for A. Intelligent Technology Cognition and Foundational Literacy was 0.1981; B. Physical Education Digital Tools and Data Literacy was 0.3032; C. Digitalized Physical Education Teaching Integration Competence was 0.3474; D. Professional Development and Social Impact was 0.1513. The consistency check results showed a CR = 0.0038 < 0.1, as detailed in Supplementary Table 5.

#### 3.4.2 Secondary indicator weights

**FIGURE 3:**
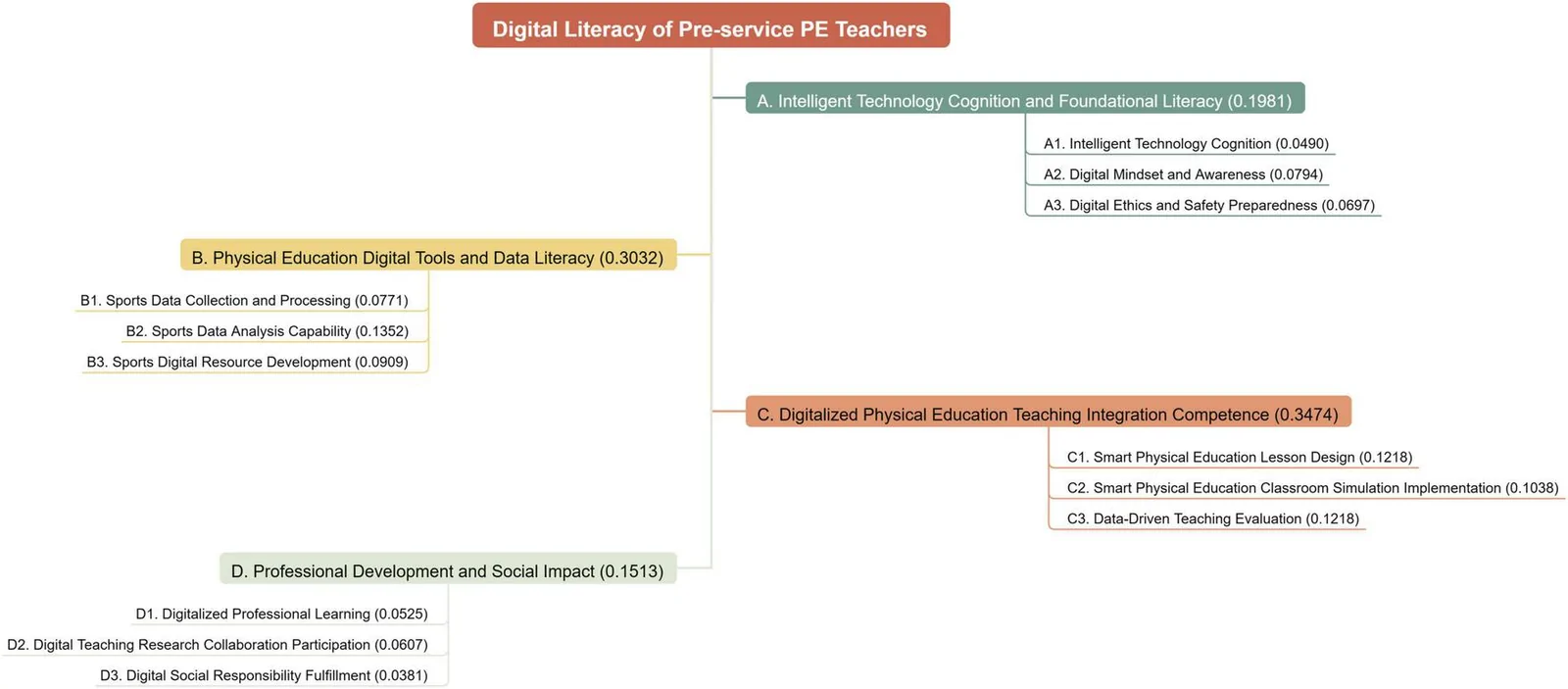
Hierarchical structure of the digital literacy assessment framework with indicator weights. Values in parentheses indicate comprehensive weights, and darker colors indicate higher weights. The framework comprises four primary dimensions, A through D, and 12 secondary indicators. Digitalized Physical Education Teaching Integration Competence (C: 0.3474) received the highest weight, followed by Physical Education Digital Tools and Data Literacy (B: 0.3032), Intelligent Technology Cognition and Foundational Literacy (A: 0.1981), and Professional Development and Social Impact (D: 0.1513).

The calculated comprehensive weights for the secondary indicators are detailed in Table 9. Ranked by comprehensive weight from highest to lowest: B2. Sports Data Analysis Capability (0.1352), C1. Smart Physical Education Lesson Design (0.1218), C3. Data-Driven Teaching Evaluation (0.1218), C2. Smart Physical Education Classroom Simulation Implementation (0.1038), B3. Sports Digital Resource Development (0.0909), A2. Digital Mindset and Awareness (0.0794), B1. Sports Data Collection and Processing (0.0771), A3. Digital Ethics and Safety Preparedness (0.0697), D2. Digital Teaching Research Collaboration Participation (0.0607), D1. Digitalized Professional Learning (0.0525), A1. Intelligent Technology Cognition (0.0490), D3. Digital Social Responsibility Fulfillment (0.0381). The hierarchical structure and weight distribution are visualized in Figure 3.

**TABLE 9:** Summary of indicator weights.

Primary indicator	Within-group weight	Secondary indicator	Within-group weight	Comprehensive weight
A. Intelligent technology cognition and foundational literacy	0.1981	A1. Intelligent technology cognition	0.2476	0.0490
		A2. Digital mindset and awareness	0.4008	0.0794
		A3. Digital ethics and safety preparedness	0.3516	0.0697
B. Physical education digital tools and data literacy	0.3032	B1. Sports data collection and processing	0.2542	0.0771
		B2. Sports data analysis capability	0.4460	0.1352
		B3. Sports digital resource development	0.2998	0.0909
C. Digitalized physical education teaching integration competence	0.3474	C1. Smart physical education lesson design	0.3507	0.1218
		C2. Smart physical education classroom simulation implementation	0.2988	0.1038
		C3. Data-driven teaching evaluation	0.3505	0.1218
D. Professional development and social impact	0.1513	D1. Digitalized professional learning	0.3472	0.0525
		D2. Digital teaching research collaboration participation	0.4011	0.0607
		D3. Digital social responsibility fulfillment	0.2517	0.0381

## 4 Discussion

### 4.1 Methodological rigor and weight distribution

The methodological rigout of this study is supported by three indicators. The expert authority coefficient (Cr = 0.825) exceeded the 0.7 threshold, confirming that experts’ judgments were grounded in both theoretical knowledge and practical experience. The 100% response rate across both Delphi rounds reflects strong expert engagement (Bell et al., 2021; Ross et al., 2014). Kendall’s W increased from 0.414 in the first round to 0.568 in the second (*p* < 0.001), indicating convergence of expert opinions—suggesting that the Delphi process successfully transformed initial disagreements into a stable consensus. This convergence is noteworthy given the multidisciplinary panel (physical education pedagogy, educational technology, and educational psychology), implying that the framework transcends disciplinary silos. All AHP consistency ratios were below 0.1, confirming that the pairwise comparison matrices were mathematically consistent (Saaty and Vargas, 1987).

The primary indicators ranked by weight as follows: Digitalized Physical Education Teaching Integration Competence (0.3474), Physical Education Digital Tools and Data Literacy (0.3032), Intelligent Technology Cognition and Foundational Literacy (0.1981), and Professional Development and Social Impact (0.1513). This ranking empirically substantiates the TPACK framework’s emphasis on technology-pedagogy-content synergy (Mishra and Koehler, 2006; Pazilah et al., 2024). While prior studies have identified technology integration as a driver of innovative teaching (Zhao et al., 2025) and technical skills as a critical dimension of digital literacy (Amemasor et al., 2025), the present findings extend this literature by quantifying the relative priority of these competencies in PE teacher education. The gap between Integration Competence (0.3474) and Foundational Cognition (0.1981) suggests that experts discriminate sharply between declarative knowledge about technology and its operationalization in teaching, a distinction that general frameworks such as DigCompEdu do not explicitly weight. The hierarchical structure moves from cognition through tool proficiency and pedagogical application to professional growth, providing a coherent logic for curriculum design in PE teacher education, to which we return in the following subsections.

### 4.2 Intelligent technology cognition and foundational literacy

Turning to the individual dimensions, primary indicator A (Intelligent Technology Cognition and Foundational Literacy) forms the cognitive and conceptual foundation of pre-service PE teachers’ digital literacy, with a weight of 0.1981 (third highest among the four dimensions). This dimension consists of three secondary indicators: A2. Digital Mindset and Awareness (0.0794), A3. Digital Ethics and Safety Preparedness (0.0697), and A1. Intelligent Technology Cognition (0.0490).

Among the secondary indicators in this dimension, A2. Digital Mindset and Awareness carried the highest composite weight (0.0794), suggesting that experts place greater value on cultivating mindset and awareness than on technical cognition alone. This finding resonates with teacher belief theory: teachers’ cognitive beliefs and affective attitudes toward technology predict technology integration behavior far more strongly than declarative technical knowledge alone (Scherer et al., 2019). From an SDT perspective, cultivating a digital mindset essentially satisfies pre-service PE teachers’ psychological need for competence in digital technology application; only when they perceive themselves as capable of effectively using digital tools in PE teaching can they form a positive attitude and reduce techno-anxiety (Lutsenko et al., 2025). The second-highest weight in this dimension was assigned to A3. Digital Ethics and Safety Preparedness (0.0697), reflecting growing concern over student data privacy and cybersecurity—prerequisites for responsible technology use. This also captures the moral-psychological dimension of digital literacy (Haynes and Robinson, 2023; Steinerová, 2023). The comparatively lower weight of A1. Intelligent Technology Cognition (0.0490) suggests that declarative knowledge of what technology is matters less than how teachers think about it (mindset) and how they act on it (ethical principles). This pattern is consistent with TAM’s core premise—perceived usefulness and ease of use are stronger predictors of technology adoption than mere factual knowledge (Scherer et al., 2019). From an SDT perspective, the high weight of Digital Mindset (0.0794) reflects the competence need: when pre-service teachers perceive themselves as capable of using digital tools effectively in PE, they are more likely to internalize technology as part of their professional identity (Sun et al., 2017). Thus, the dimension’s weight structure empirically validates that motivational beliefs outweigh declarative knowledge in shaping digital literacy.

### 4.3 Physical education digital tools and data literacy

With a weight of 0.3032—the second highest among the four dimensions—primary indicator B(Physical Education Digital Tools and Data Literacy) constitutes the core tool-based foundation of pre-service PE teachers’ digital literacy. Among the three secondary indicators in this dimension, B2. Sports Data Analysis Capability (0.1352)received the highest weight of any secondary indicator, underscoring the central role of data analysis in physical education. This finding is in line with broader trends in educational big data and precision teaching (Zhong et al., 2025). From a cognitive psychology perspective, sports data analysis competence is a high-level metacognitive activity that involves perceiving, encoding, comparing, and reasoning about students’ motor performance and exercise load data (Petko et al., 2025)—the cognitive core of data-driven instruction in PE. B1. Sports Data Collection and Processing (0.0771), a newly added indicator, serves as the foundational cognitive link in this metacognitive process; without proficient data collection, the subsequent metacognitive analysis cannot be effectively performed. B3. Sports Digital Resource Development (0.0909) blends cognitive psychology with constructivist learning theory: creating personalized digital resources based on students’ cognitive characteristics embodies cognitive constructivism in digital PE practice (Gao et al., 2025). This dimension’s weight (0.3032) also reflects TAM’s perceived usefulness mechanism: when teachers recognize that data analysis and digital resources directly improve teaching effectiveness and student learning outcomes, their intention to adopt these tools increases (Phelps et al., 2021). The high weight of B2 (Sports Data Analysis Capability, 0.1352) further confirms that metacognitive skills—perceiving, encoding, and reasoning about student performance data—are valued as the cognitive core of data-driven PE instruction (Alhadad, 2018).

### 4.4 Digitalized physical education teaching integration competence

Carrying the highest weight of the four dimensions (0.3474), primary indicator C(Digitalized Physical Education Teaching Integration Competence) represents the core and ultimate objective of pre-service PE teachers’ digital literacy. The three secondary indicators within this dimension—C1. Smart Physical Education Lesson Design (0.1218), C3. Data-Driven Teaching Evaluation (0.1218), and C2. Smart Physical Education Classroom Simulation Implementation (0.1038)—all received high weights, underscoring the centrality of teaching practice integration competence in the expert consensus.

C1. Smart Physical Education Lesson Design and C3. Data-Driven Teaching Evaluation shared the highest composite weights (0.1218 each), indicating that lesson design and teaching evaluation are equally critical. This equal weight reflects both the TPACK framework and teacher digital teaching self-efficacy theory (Tian et al., 2024). Digital teaching self-efficacy—the confidence to design smart PE lessons and conduct data-driven evaluations—is a key psychological variable shaping actual digital teaching behavior (Huang et al., 2025). C2. Smart Physical Education Classroom Simulation Implementation (0.1038) is the behavioral embodiment of this self-efficacy, linking cognitive lesson design to classroom practice. The high weight confirms that translating cognitive self-efficacy into tangible teaching actions lies at the heart of digital literacy development (Semiz and Ince, 2012; Yu and Alibakhshi, 2025).

The high weight of Integration Competence (0.3474) can be further interpreted through the lens of the Technology Acceptance Model (TAM) and Self-Determination Theory (SDT). From a TAM perspective, the emphasis on lesson design and data-driven evaluation reflects teachers’ perceived usefulness of digital tools—when teachers believe that digital technology can improve student learning outcomes in PE, they are more likely to adopt it (Scherer et al., 2019). From an SDT perspective, the high weight of this dimension also satisfies teachers’ need for competence: designing and implementing digital-enhanced PE lessons provides mastery experiences that build confidence in technology use (Sun et al., 2017).

#### 4.4.1 Implications for curriculum design and teacher preparation

The weight distribution of the framework offers practical guidance for curriculum design in pre-service PE teacher education programs. First, the emphasis on Digitalized Physical Education Teaching Integration Competence (0.3474) suggests that training programs should prioritize practice-based learning experiences, such as micro-teaching sessions that require pre-service teachers to design and deliver technology-enhanced PE lessons in simulated or real classroom settings (Tondeur et al., 2017). Second, the high weight assigned to Physical Education Digital Tools and Data Literacy (0.3032) indicates that curricula should include dedicated modules on wearable device operation, sports performance data analysis, and digital resource development, equipping future teachers with the technical skills needed for data-driven instruction (Zhao and Su, 2026). Third, given the relatively lower weight of Professional Development and Social Impact (0.1513), this dimension should not be neglected but rather integrated longitudinally across the program, fostering collaborative research engagement and ethical digital citizenship through sustained professional learning communities rather than isolated workshops (Tondeur et al., 2017).

Despite the framework’s theoretical and practical value, several challenges may arise when institutions seek to implement it. First, resource constraints—including limited access to smart devices, wearable sensors, and data analytics platforms—may hinder the practical application of the framework, particularly in under-resourced educational settings (Østerlie et al., 2025). Second, the varying levels of digital competence among teacher educators themselves may affect the quality of instruction and mentoring provided to pre-service teachers (Blannin et al., 2026). Third, institutional resistance to curriculum change and the absence of supportive policies or incentives for technology-integrated teaching may slow adoption (Zhao et al., 2025). Addressing these challenges will require institutional commitment, ongoing professional development for both pre-service and in-service teachers, and strategic investment in digital infrastructure (Østerlie et al., 2025; Tondeur et al., 2017).

### 4.5 Professional development and social impact

With the lowest weight among the four dimensions (0.1513), primary indicator D(Professional Development and Social Impact) represents the dimension of sustainable development motivation and social responsibility within pre-service PE teachers’ digital literacy. The three secondary indicators in this dimension were weighted as follows: D2. Digital Teaching Research Collaboration Participation (0.0607), D1. Digitalized Professional Learning (0.0525), and D3. Digital Social Responsibility Fulfillment (0.0381).

Among these, D2. Digital Teaching Research Collaboration Participation carried the highest weight, indicating that within the professional development dimension, collaboration is viewed as a core element. This resonates with SDT’s emphasis on relatedness (MacPhail et al., 2025; Sun et al., 2017): digital teaching collaboration within online communities satisfies pre-service PE teachers’ social-psychological needs for interaction and mutual learning, thereby promoting the sustainable growth of their digital literacy. D1. Digitalized Professional Learning (0.0525) provides the cognitive foundation, essential for maintaining professional cognitive renewal in the digital age (Gorozidis et al., 2020; Tomczyk, 2024). This aligns with the broader finding by Tondeur et al. (2017) that professional development is a key enabler for pre-service teachers’ technology integration. D3. Digital Social Responsibility Fulfillment (0.0381), although the lowest in weight, represents the highest psychological dimension—prosocial moral psychology—requiring teachers to guide students toward safe and ethical technology use (Biedermann and Nagel, 2021; Scuotto et al., 2024).

In summary, the four dimensions form a coherent psychological development chain for pre-service PE teachers’ digital literacy, integrating multiple educational psychology theories. The chain begins with the cognitive-motivational foundation (SDT, TAM, teacher belief theory), moves through metacognitive training in using digital tools and processing data (cognitive psychology), then to the manifestation of digital teaching self-efficacy (TPACK, teacher self-efficacy theory), and finally arrives at the satisfaction of social-psychological needs and the development of moral psychology (SDT, digital citizenship theory). This psychological logic gives the framework both strong disciplinary relevance to PE and a universal psychological grounding for understanding teacher digital literacy development. To further contextualize the proposed framework, a comparison of its four dimensions with the corresponding areas of the DigCompEdu and UNESCO ICT-CFT frameworks is provided in Supplementary Table 6. The comparison highlights both commonalities—such as the emphasis on digital awareness, tool operation, and professional development—and PE-specific differentiators, including the focus on sports performance data analysis, wearable device application, and simulation-based teaching in physical education contexts.

### 4.6 Limitations

Although the digital literacy assessment framework for pre-service PE teachers developed in this study is both scientifically rigorous and practical, several limitations should be acknowledged.

Second, the Delphi method and the analytic hierarchy process (AHP) inherently rely on subjective expert judgments. While we employed multiple consultation rounds and consistency checks to minimize bias, the potential influence of subjectivity cannot be completely eliminated. Incorporating empirical approaches—such as large-scale surveys or classroom observations—would help validate and refine the framework (Kotera et al., 2025; Sokolov et al., 2025). Third, this study focused on framework construction and weight determination; the framework has not yet been empirically validated with actual pre-service PE teachers. Future research should translate the indicator system into a standardized measurement scale and conduct exploratory factor analysis (EFA) to uncover the underlying factor structure, followed by confirmatory factor analysis (CFA) to test the hypothesized four-dimensional model. Reliability should be assessed using Cronbach’s alpha and composite reliability, and measurement invariance should be tested across different subgroups (e.g., gender, teaching level) to ensure generalizability. Criterion-related validity should also be examined by correlating scale scores with external criteria such as teaching performance ratings or technology integration self-reports. Only after such empirical testing can the framework be confidently applied in teacher education programs.

## 5 Conclusion

This study employed the Delphi method and the Analytic Hierarchy Process to develop a digital literacy assessment framework for pre-service physical education teachers, comprising 4 primary indicators and 12 secondary indicators. The research results indicate that the framework possesses high scientific rigor and reliability. Weight analysis revealed that Digitalized Physical Education Teaching Integration Competence (0.3474) and Physical Education Digital Tools and Data Literacy (0.3032) are the core dimensions. The most critical secondary competencies are Sports Data Analysis Capability (0.1352), Smart Physical Education Lesson Design (0.1218), and Data-Driven Teaching Evaluation (0.1218). This framework can serve as a target-oriented guide for the cultivation of pre-service physical education teachers and provide a theoretical basis for curriculum reform in physical education teacher preparation programs. As an assessment framework, it is intended to provide a structured approach for evaluating pre-service PE teachers’ digital literacy development, rather than a prescriptive curriculum model or a standalone competency list. In addition, its underlying psychological development chain offers a new perspective for psychological research on teacher digital literacy in other disciplines.

## Data Availability

The original contributions presented in the study are included in the article/Supplementary material, further inquiries can be directed to the corresponding author.
