# Supplementary material for: Developing and validating a digital literacy assessment framework for pre-service physical education teachers: a Delphi-AHP study
Source: Front Psychol. 2026 Aug 20;17:1852701. doi: 10.3389/fpsyg.2026.1852701 (PMC13538841; doi:10.3389/fpsyg.2026.1852701)
Supplement: Supplementary file 1 [file Table_3.DOCX]

**Table S1.** Initial candidate indicators and sources

| Code | Initial Candidate Indicator | Source Literature / Policy Framework | Final Status |
| --- | --- | --- | --- |
| A1 | Intelligent Technology Cognition | TPACK framework (Mishra & Koehler, 2006); DigCompEdu framework (Redecker, 2017) | Retained |
| A2 | Digital Mindset and Awareness | Technology Acceptance Model (Davis, 1989); Scherer et al. (2019) | Retained |
| A3 | Digital Ethics and Safety Preparedness | Haynes & Robinson (2023); Steinerová (2023); Biedermann & Nagel (2021) | Retained |
| B1 | Smart Sports Equipment Operation | Literature review; UNESCO (2018); Zhao & Su (2026) | Deleted |
| B11 | Sports Data Collection and Processing | Expert suggestion (based on wearable device trends; Zhao & Su, 2026) | Added |
| B2 | Sports Data Analysis Capability | Petko et al. (2025); Alhadad (2018); Zhong et al. (2025) | Retained |
| B3 | Sports Digital Resource Development | Gao et al. (2025); Wohlfart et al. (2024) | Retained |
| C1 | Smart Physical Education Lesson Design | TPACK framework (Mishra & Koehler, 2006); Tian et al. (2024) | Retained |
| C2 | Smart Physical Education Classroom Simulation Implementation | Semiz & Ince (2012); Yu & Alibakhshi (2025); Tondeur et al. (2017) | Retained |
| C3 | Data-Driven Teaching Evaluation | Huang et al. (2025); Tian et al. (2024) | Retained |
| D1 | Digitalized Professional Learning | Gorozidis et al. (2020); Tomczyk (2024); Zhang et al. (2026) | Retained |
| D2 | Digital Teaching Research Collaboration Participation | Self-Determination Theory (Sun et al., 2017; MacPhail et al., 2025) | Retained |
| D3 | Digital Social Responsibility Fulfillment | Scuotto et al. (2024); Biedermann & Nagel (2021); Sun et al. (2017) | Retained |

Initial candidate indicators were derived from all frameworks and key literature cited in the literature review. B1 was deleted for failing to meet the retention criteria, and B11 was added based on expert suggestions, as noted in the table.

**Table S2.** Summary of indicator changes across Delphi rounds

| Code | Indicator Name | Round 1 Mean | Round 1 CV | Round 1 Full-Score Freq. | Round 1 Status | Round 2 Mean | Round 2 CV | Round 2 Full-Score Freq. | Round 2 Status | Reason for Change / Expert Feedback Example |
| --- | --- | --- | --- | --- | --- | --- | --- | --- | --- | --- |
| A1 | Intelligent Technology Cognition | 3.9 | 0.2185 | 0.30 | Retained | 4.1 | 0.2224 | 0.45 | Retained | — |
| A2 | Digital Mindset and Awareness | 4.7 | 0.1398 | 0.80 | Retained | 4.85 | 0.0755 | 0.85 | Retained | “Teachers’ beliefs about technology are more important than technical knowledge alone” |
| A3 | Digital Ethics and Safety Preparedness | 4.6 | 0.1639 | 0.75 | Retained | 4.7 | 0.1215 | 0.75 | Retained | “Data privacy and cybersecurity are prerequisites for responsible technology use in schools” |
| B1 | Smart Sports Equipment Operation | 3.35 | 0.2612 | 0.10 | Deleted | — | — | — | — | Failed all three criteria: mean<3.5, CV>0.25, full-score<0.2. Expert feedback: “The focus should be on data utilisation rather than equipment operation alone” |
| B11 | Sports Data Collection and Processing | — | — | — | Added | 4.55 | 0.1122 | 0.55 | Retained | “The proliferation of wearable devices and motion sensors in PE requires data collection skills” |
| B2 | Sports Data Analysis Capability | 4.65 | 0.1443 | 0.75 | Retained | 4.1 | 0.1922 | 0.35 | Retained | “Data analysis is the cognitive core of data-driven PE instruction” |
| B3 | Sports Digital Resource Development | 4.05 | 0.2190 | 0.40 | Retained | 4.95 | 0.0452 | 0.95 | Retained | “Creating digital resources is a key competence for modern PE teachers” |
| C1 | Smart Physical Education Lesson Design | 4.0 | 0.1814 | 0.25 | Retained | 4.9 | 0.0628 | 0.90 | Retained | “Lesson design should integrate digital tools with pedagogical goals” |
| C2 | Smart Physical Education Classroom Simulation Implementation | 4.65 | 0.1443 | 0.75 | Retained | 3.95 | 0.1922 | 0.25 | Retained | Wording refined to specify “simulated or real practicum settings” based on expert feedback: “Pre-service teachers need practice in authentic teaching contexts” |
| C3 | Data-Driven Teaching Evaluation | 4.8 | 0.0855 | 0.80 | Retained | 3.8 | 0.2193 | 0.25 | Retained | — |
| D1 | Digitalized Professional Learning | 3.85 | 0.2273 | 0.30 | Retained | 4.75 | 0.1158 | 0.80 | Retained | “Digital professional development should be a lifelong learning process” |
| D2 | Digital Teaching Research Collaboration Participation | 3.85 | 0.2273 | 0.30 | Retained | 4.9 | 0.0628 | 0.90 | Retained | “Collaboration within online communities satisfies teachers’ social-psychological needs” |
| D3 | Digital Social Responsibility Fulfillment | 4.65 | 0.1443 | 0.75 | Retained | 4.1 | 0.2079 | 0.40 | Retained | “PE teachers should guide students toward safe and ethical technology use” |

(1) Retention criteria: Round 1 – ≥80% of experts rated the indicator ≥4; Round 2 – mean > 3.5, CV < 0.25, full-score frequency > 0.2, all three criteria required.

(2) B11 was introduced in Round 2 based on expert suggestions and was therefore not evaluated in Round 1.

**Table S3.**  List of Delphi experts and individual consistency ratios (CR)

| No. | Name | Title¹ | YOPE² | Research Field | Institution (Region) | λmax | CI | CR | CR < 0.1 |
| --- | --- | --- | --- | --- | --- | --- | --- | --- | --- |
| 1 | Zhang ** | Professor | 30 | Physical Education | Hanjiang Normal University (Hubei, Central China) | 4.2454 | 0.0818 | 0.0919 | Yes |
| 2 | Liu * | Professor | 24 | Educational Technology | Central China Normal University (Hubei, Central China) | 4.2217 | 0.0739 | 0.0830 | Yes |
| 3 | Wang ** | Professor | 32 | Physical Education | Wuhan Sports University (Hubei, Central China) | 4.2465 | 0.0822 | 0.0924 | Yes |
| 4 | Chen * | Associate Professor | 28 | Physical Education | Hubei University (Hubei, Central China) | 4.1622 | 0.0541 | 0.0608 | Yes |
| 5 | Li ** | Professor | 22 | Educational Psychology | Huazhong University of Science and Technology (Hubei, Central China) | 4.1053 | 0.0351 | 0.0394 | Yes |
| 6 | Yang * | Associate Professor | 36 | Physical Education | Hanjiang Normal University (Hubei, Central China) | 4.1171 | 0.0390 | 0.0438 | Yes |
| 7 | Zhao ** | Professor | 20 | Physical Education | Nanjing Sport Institute (Jiangsu, East China) | 4.1279 | 0.0426 | 0.0479 | Yes |
| 8 | Wu * | Professor | 28 | Educational Technology | South China Normal University (Guangdong, South China) | 4.1379 | 0.0460 | 0.0517 | Yes |
| 9 | Huang ** | Associate Professor | 14 | Physical Education | Beijing Sport University (Beijing, North China) | 4.1384 | 0.0461 | 0.0518 | Yes |
| 10 | Zhou * | Professor | 30 | Physical Education | Shanghai University of Sport (Shanghai, East China) | 4.1854 | 0.0618 | 0.0694 | Yes |
| 11 | Xu ** | Associate Professor | 12 | Educational Psychology | Nanjing Normal University (Jiangsu, East China) | 4.1824 | 0.0608 | 0.0683 | Yes |
| 12 | Sun * | Associate Professor | 20 | Educational Technology | Northeast Normal University (Jilin, Northeast China) | 4.0455 | 0.0152 | 0.0171 | Yes |
| 13 | Ma ** | Professor | 28 | Educational Technology | Shaanxi Normal University (Shaanxi, Northwest China) | 4.2414 | 0.0805 | 0.0904 | Yes |
| 14 | Zhu * | Professor | 13 | Educational Technology | Chongqing Normal University (Chongqing, Southwest China) | 4.1023 | 0.0341 | 0.0383 | Yes |
| 15 | Lin ** | Associate Professor | 11 | Educational Psychology | Zhejiang University (Zhejiang, East China) | 4.1147 | 0.0382 | 0.0429 | Yes |
| 16 | He * | Associate Professor | 22 | Educational Psychology | Sichuan Normal University (Sichuan, Southwest China) | 4.2231 | 0.0744 | 0.0836 | Yes |
| 17 | Gao ** | Associate Professor | 15 | Physical Education | Hunan Normal University (Hunan, Central China) | 4.2430 | 0.0810 | 0.0910 | Yes |
| 18 | Luo * | Associate Professor | 25 | Educational Technology | Jiangxi Normal University (Jiangxi, East China) | 4.0729 | 0.0243 | 0.0273 | Yes |
| 19 | Xiao ** | Associate Professor | 12 | Physical Education | Fujian Normal University (Fujian, East China) | 4.0457 | 0.0152 | 0.0171 | Yes |
| 20 | Peng * | Associate Professor | 31 | Physical Education | Northwest Normal University (Gansu, Northwest China) | 4.2367 | 0.0789 | 0.0887 | Yes |

1.All professional titles are standardised based on the commonly used academic rank system in China: Associate Professor and Professor.

2.YOPE = Years of professional experience (calculated as of April 1, 2026).

**Table S4.** Summary of Consistency Ratios (CR) for All Matrices

| Matrix Group | Number of Matrices | Acceptable CR (≤0.10) | CR > 0.10 (Corrected) | CR > 0.10 (Excluded) | Final Included |
| --- | --- | --- | --- | --- | --- |
| Primary Indicators (4 dimensions) | 20 | 20 | 0 | 0 | 20 |
| Dimension A (A1–A3) | 20 | 20 | 0 | 0 | 20 |
| Dimension B (B1–B3) | 20 | 20 | 0 | 0 | 20 |
| Dimension C (C1–C3) | 20 | 20 | 0 | 0 | 20 |
| Dimension D (D1–D3) | 20 | 20 | 0 | 0 | 20 |
| Total | 100 | 100 | 0 | 0 | 100 |

All consistency ratios (CR) were below 0.1, meeting the acceptable threshold recommended by Saaty and Vargas (1987). No matrices were excluded from the final analysis.

Dimension A = Intelligent Technology Cognition and Foundational Literacy; B = Physical Education Digital Tools and Data Literacy; C = Digitalized Physical Education Teaching Integration Competence; D = Professional Development and Social Impact.

**Table S5** Integrated judgment matrix and weights for primary indicators.

|  | A | B | C | D | Within-Group Weight |
| --- | --- | --- | --- | --- | --- |
| **A** | 1 | 0.6431 | 0.5317 | 1.4255 | **0.1981** |
| **B** | 1.555 | 1 | 0.8117 | 2.1216 | **0.3032** |
| **C** | 1.8809 | 1.2319 | 1 | 1.9908 | **0.3474** |
| **D** | 0.7015 | 0.4714 | 0.5023 | 1 | **0.1513** |

A=Intelligent Technology Cognition and Foundational Literacy;B=Physical Education Digital Tools and Data Literacy;C=Digitalized Physical Education Teaching Integration Competence;D=Professional Development and Social Impact.

**Table S6.** Comparison of the proposed framework with DigCompEdu and UNESCO ICT-CFT

| Proposed Framework Dimension | Corresponding DigCompEdu Area | Corresponding UNESCO ICT-CFT Module | Similarities | PE-Specific Differences |
| --- | --- | --- | --- | --- |
| A. Intelligent Technology Cognition and Foundational Literacy | Digital literacy foundation | Technology literacy | All emphasise basic digital awareness and cognition | Emphasises technology awareness specific to PE teaching contexts (e.g., smart devices in sports) |
| B. Physical Education Digital Tools and Data Literacy | Digital resources / Assessment | Knowledge deepening | All include digital tool operation and data handling | Highlights sports performance data analysis and wearable device application |
| C. Digitalized Physical Education Teaching Integration Competence | Teaching and learning | Knowledge creation | All emphasise technology-pedagogy integration | Focuses on PE classroom contexts (movement instruction, simulation-based teaching) |
| D.Professional Development and Social Impact | Empowering learners' digital competence | Professional learning | All include continuous professional development | Emphasises PE-specific ethical responsibilities (student safety, health data privacy) |
